# The Membrane Protein Sortilin Is a Potential Biomarker and Target for Glioblastoma

**DOI:** 10.3390/cancers15092514

**Published:** 2023-04-27

**Authors:** Mark Marsland, Amiee Dowdell, Sam Faulkner, Craig Gedye, James Lynam, Cassandra P. Griffin, Joanne Marsland, Chen Chen Jiang, Hubert Hondermarck

**Affiliations:** 1 School of Biomedical Sciences and Pharmacy, College of Health, Medicine and Wellbeing, University of Newcastle, Callaghan, NSW 2308, Australia; mark.marsland@uon.edu.au (M.M.); amiee.dowdell@uon.edu.au (A.D.); sam.faulkner@newcastle.edu.au (S.F.); cassandra.griffin@newcastle.edu.au (C.P.G.); joanne.marsland@uon.edu.au (J.M.); chenchen.jiang@newcastle.edu.au (C.C.J.); 2 Hunter Medical Research Institute, University of Newcastle, New Lambton Heights, NSW 2305, Australia; craig.gedye@calvarymater.org.au (C.G.); james.lynam@calvarymater.org.au (J.L.); 3 School of Medicine and Public Health, College of Health, Medicine and Wellbeing, University of Newcastle, Callaghan, NSW 2308, Australia; 4 Department of Medical Oncology, Calvary Mater, Newcastle, NSW 2298, Australia; 5 Hunter Cancer Biobank, NSW Regional Biospecimen and Research Services, University of Newcastle, Callaghan, NSW 2305, Australia

**Keywords:** Glioblastoma, sortilin, cancer biomarkers, cancer therapeutic targets

## Abstract

**Simple Summary:**

Glioblastoma (GBM) is the most lethal adult primary brain tumor, and has no cure. This study investigated the membrane protein sortilin as a prognosis biomarker for glioblastoma (GBM). We found that sortilin is overexpressed in GBM tumors and can be detected in the blood of GBM patients. In addition, in cell cultures, targeting sortilin resulted in the inhibition of GBM cell invasion. These data highlight the value of sortilin as a potential clinical biomarker and therapeutic target for GBM and warrant further translational investigation.

**Abstract:**

Glioblastoma (GBM) is a devastating brain cancer with no effective treatment, and there is an urgent need for developing innovative biomarkers as well as therapeutic targets for better management of the disease. The membrane protein sortilin has recently been shown to participate in tumor cell invasiveness in several cancers, but its involvement and clinical relevance in GBM is unclear. In the present study, we explored the expression of sortilin and its potential as a clinical biomarker and therapeutic target for GBM. Sortilin expression was investigated by immunohistochemistry and digital quantification in a series of 71 clinical cases of invasive GBM vs. 20 non-invasive gliomas. Sortilin was overexpressed in GBM and, importantly, higher expression levels were associated with worse patient survival, pointing to sortilin tissue expression as a potential prognostic biomarker for GBM. Sortilin was also detectable in the plasma of GBM patients by enzyme-linked immunosorbent assay (ELISA), but no differences were observed between sortilin levels in the blood of GBM vs. glioma patients. In vitro, sortilin was detected in 11 brain-cancer-patient-derived cell lines at the anticipated molecular weight of 100 kDa. Interestingly, targeting sortilin with the orally bioavailable small molecule inhibitor AF38469 resulted in decreased GBM invasiveness, but cancer cell proliferation was not affected, showing that sortilin is targetable in GBM. Together, these data suggest the clinical relevance for sortilin in GBM and support further investigation of GBM as a clinical biomarker and therapeutic target.

## 1. Introduction

Glioblastoma multiforme (GBM) is the most common and lethal malignant primary brain tumor in adults, accounting for 45% of brain cancer cases [1], with a median survival between 7 and 15 months [2]. This poor prognosis is due to the aggressive and invasive nature of GBM and the absence of effective targeted treatment [3]. The oral alkylating agent temozolomide (TMZ) is the standard first-line chemotherapy [4]. Unfortunately, resistance to TMZ and recurrence of GBM are inevitable, and this is particularly dramatic in the cases of GBM exhibiting unmethylated O6-methylguanine-DNA methyltransferase (MGMT); only 7% of GBM patients with this epigenetic silencing survive 5 years or longer [5]. Therefore, the identification of new therapeutic targets for GBM is necessary for the design of effective targeted treatment that could improve the currently limited efficacy of TMZ.

Sortilin (SORT1), also known as neurotensin receptor-3 (NTR3), is a membrane receptor that belongs to the VPS10P (vacuolar protein sorting 10 protein) family of receptors [6]. The biological roles of sortilin include sorting and transporting intracellular proteins. Sortilin has been associated with the progression and aggressiveness of several malignancies, including liver cancer [7,8], pancreatic cancer [9], breast cancer [10,11], metastatic melanoma [12], and colorectal cancer [13]. In GBM cells, sortilin promotes invasion and mesenchymal transition through a mechanism involving a GSK-3β/β-catenin/twist pathway [14] and presenilin1 [15], but the relevance of sortilin as a clinical biomarker or a therapeutic target is unclear.

In this study, we have explored the clinical relevance of sortilin in GBM. Using GBM patient samples and patient-derived cells, we have shown that sortilin expression is elevated in GBM compared to lower-grade glioma, and that sortilin was also detectable at varying concentrations in the blood of GBM and lower-grade glioma patients. In addition, sortilin inhibition was able to inhibit the invasion of GBM cells. These data point to sortilin as a potential biomarker and therapeutic target for GBM.

## 2. Materials and Methods

### 2.1. SORT1 (Sortilin) mRNA Data Mining

Gene Expression Profiling Interactive Analysis 2 (GEPIA2) (http://gepia.cancer-pku.cn (accessed on 1 December 2022)) was used to explore GBM data in TCGA [16] and normal brain tissue in Genotype-Tissue Expression (GTEx) [17] databases, using a standard processing pipeline [18]. SORT1 mRNA expressions in GBM, lower-grade glioma (LGG) and normal brain tissue were compared in terms of survival analysis in GBM and LGG comparing high (>median) vs. low (<median) gene expression of *SORT1*. One-way ANOVA was used for differential analysis of gene expression, using disease states (GBM, LGG or normal) as variables for the box plots. Log-rank tests for both disease-free survival and overall survival analyses were used.

### 2.2. Patient Samples

Patient cohort information included age, sex, tumor grade and primary tumor site (Table 1). Tumor samples were sourced from the Hunter Cancer Biobank (HCB, Newcastle, NSW, Australia) and then formalin-fixed paraffin-embedded (FFPE). All samples were graded by a clinical pathologist from the HCB using the clinically relevant histological features in the WHO guidelines used in clinical practice. The study was approved by the Human Research Ethics Committee of the University of Newcastle. Tumor samples included 71 cases of GBM, 12 cases of grade 3 glioma, 6 cases of grade 2 glioma, and 2 cases of grade 1 glioma. Matching plasma samples were obtained at the time of diagnosis and processed following standard clinical procedure for plasma, including centrifugation for 15 min at 1500 RPM, followed by 10 min at 2500 RPM. Samples were stored at −80 °C.

### 2.3. Immunohistochemical Detection and Quantification of Sortilin Expression

FFPE tissue sections of 4 µm were processed for sortilin immunohistochemical detection as previously described [19]. Sections were labelled with anti-sortilin (0.8 mg/mL, catalogue number ANT-009, Alomone labs, Jerusalem, Israel) followed by a secondary antibody (catalogue number MP-7401, Vector Laboratories, Newark, CA, USA). Following IHC, slides were digitized using the Aperio AT2 scanner (Leica Biosystems, Wetzlar, Germany) at 40× absolute resolution. Quantification of sortilin immunohistological staining intensities was performed using the HALOTM image analysis platform (version 3.3, Indica Labs, Albuquerque, NM, USA), as reported [19].

### 2.4. Sortilin Quantification in Patient Plasma Samples

Sortilin plasma concentration was determined by Enzyme-Linked ImmunoSorbent Assay (ELISA). The ELISA kit (catalogue number SK00472-01) was from Aviscera Bioscience (Santa Clara, CA, USA). The assays were performed as recommended by the manufacturer and as previously described [20]. The Wilcoxon Rank Sum or Kruskal–Wallis (for multiple comparisons) tests were used to study the distribution of concentrations. For the primary hypothesis (differential sortilin expression between pathological subtypes), a two-sided alpha of 0.05 was employed. Statistical analyses were performed on complete cases using Prism (version 8.2.0, GraphPad Software).

### 2.5. Cell lines and Culture Conditions

Glioblastoma cancer cell lines A172 (CRL-1620) and U87MG (HTB-14) were obtained from the American Type Culture Collection (ATCC, Manassas, VA, USA). Patient-derived GBM cell lines BAH1, MN1, WK1, RN1, RKI1, HW1, PB1, SB2b, and SJH1 were a generous gift from Dr Bryan Day (QIMR Berghofer Medical Research Institute, Brisbane, QLD, Australia). Human astrocytes (HA) were from ScienCell Research Laboratories (Wangara, WA, Australia (catalogue number 1800). GBM cell lines and patient-derived GBM cell lines, including their MGMT methylation status, have been described previously [21,22]. The cell culture conditions have been described previously [19].

### 2.6. Western Blotting

Conditions for protein extraction and Western blotting were as previously published [23] with an anti-sortilin antibody (catalogue number ANT-009, Alomone labs, Israel) used at a dilution of 1:300. Also, a β-actin antibody (catalogue number A1978, Sigma-Aldrich, St. Louis, MO, USA) was used for testing equal loading at a 1:5000 dilution.

### 2.7. Measurement of Cell Growth and Invasions

Cell growth assays were carried out using Cell Titer-Blue^®^ (Promega, Hawthorne, VIC, Australia) according to the manufacturer’s instructions and as previously reported [24]. Cells were treated with AF38469 (400 nM, catalogue number HY-12802, MedChemExpress, Monmouth Junction, NJ, USA), TMZ (50 μM, catalogue number S1237, Selleck Chem, Sapphire Bioscience, NSW, Australia), or AF38469 + TMZ for 72 h. Vehicle control was DMSO at the same concentration.

Invasion assays were carried out using the 6.5 mm Transwell^®^ 8.0 µm Pore Polycarbonate Membrane Insert (Corning^®^, Sigma-Aldrich) as previously described [24]. Cells were treated with AF38469 (400 nM, catalogue number HY-12802, MedChemExpress, Monmouth Junction, NJ, USA). DMSO at the same concentration was used as vehicle control. For standard GBM cells U87MG and A172, cell invasion was quantified after 24 h, whereas the patient-derived cell lines (BAH1, RKI1, PB1) were quantified after 72 h.

### 2.8. Statistics

GraphPad Prism (La Jolla, CA, USA) was used. H-scores were analyzed as continuous variables, with summary statistics presented as group-level medians and interquartile ranges (IQR). Student’s *t*-test with unpaired two-sided was used for single comparisons. One-way analysis of variance (ANOVA) or two-way analysis of variance with Dunnett’s or Tukey’s correction were used for multiple comparisons. When data was not normally distributed, the non-parametric Kruskal–Wallis test was performed. Pearson’s correlation test was used to determine correlations. A *p* value less than 0.05 was deemed statistically significant. All experiments were performed at least in triplicate. All materials used and results generated were included for statistical analyses, with no exclusion of data points. All data are included in this publication and are presented as mean ± standard deviation (SD).

## 3. Results

### 3.1. SORT1 (Sortilin) mRNA Expression Is Not Increased in GBM Tissues

**Figure 1:**
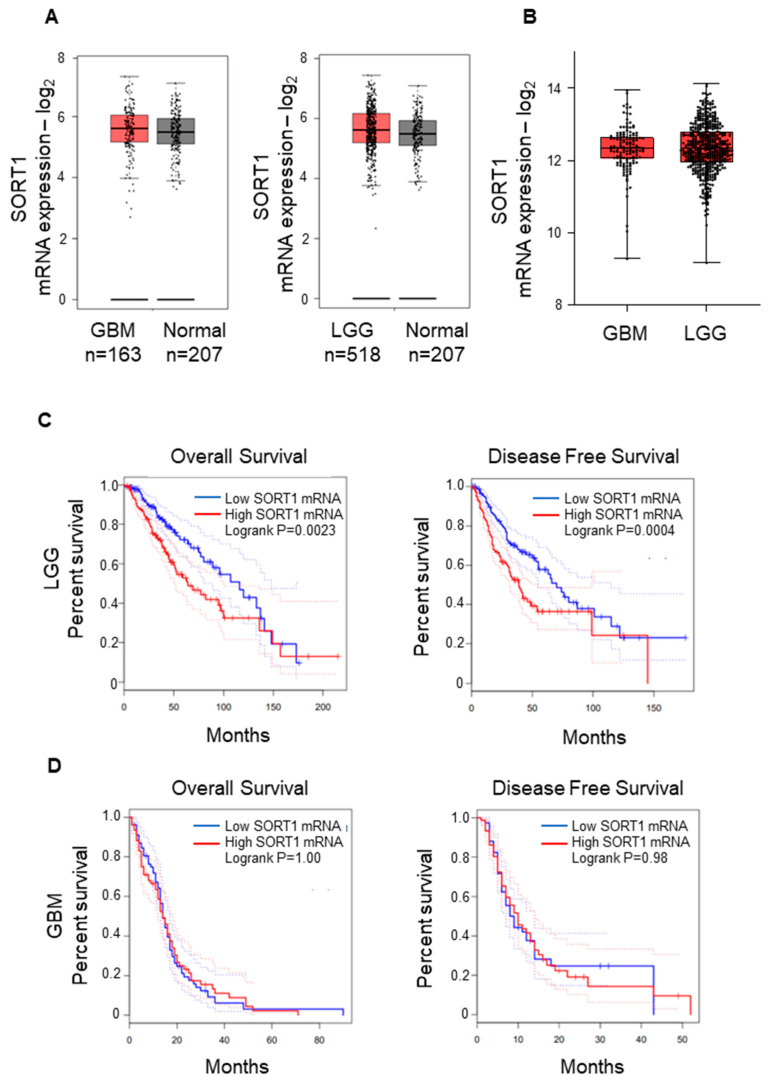
SORT1 (sortilin) mRNA expression in GBM and LGG vs. normal tissue. (**A**) The expression of SORT1 mRNA in GBM (left) and LGG (right) tissue was comparable to the expression in normal brain tissue. For normal brain tissue, GEPIA2 sourced the GTEx project (https://gtexportal.org/home/ (accessed on 1 December 2022)). (**B**) There was no observable difference between GBM and LGG SORT1 mRNA expression. (**C**) The overall survival (left) and disease-free survival (right) analysis for SORT1 mRNA in LGG revealed longer survival time for LGG patients with low SORT1 tumoral mRNA expression compared to those with high SORT1 mRNA expression (*p* = 0.0023 and *p* = 0.0004 respectively). (**D**) Analysis of overall survival (left) and disease-free survival (right) for SORT1 mRNA in GBM determined by GEPIA2.

We first performed a data mining of sortilin gene (SORT1) expression using GEPIA2 [18] and accessing the GBM and Low-Grade Glioma (LGG) datasets of The Cancer Genome Atlas (TCGA) [16] database and GTEx [25]. While there was a wide range of SORT1 mRNA expression, there was no significant difference found between the GBM, LGG and normal groups (Figure 1A,B). Interestingly, there was significant differences in LGG patients’ overall and disease-free survival comparing low and high SORT1 mRNA expression (*p* = 0.0023 and *p* = 0.0004 respectively): patients with low SORT1 expression survived longer (Figure 1C). However, this was not observed in GBM patients (Figure 1D).

### 3.2. Sortilin Protein Expression Is Increased in GBM Tissues Compared to Grade 1–3 Glioma

**Figure 2:**
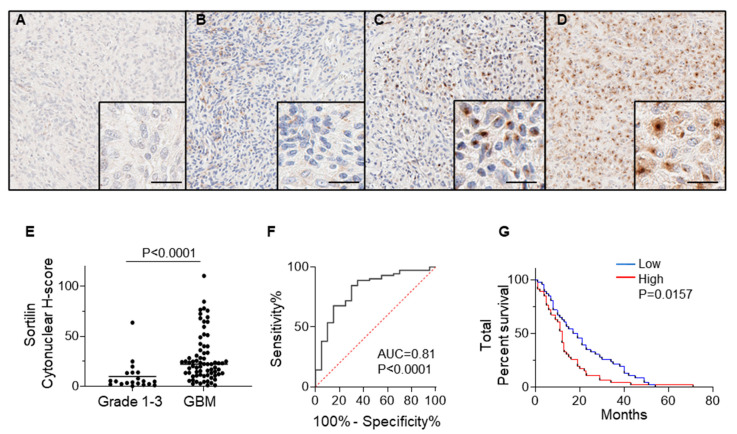
SORT1 Sortilin expression is increased in GBM vs. low grade gliomas. Representative pictures for the immunohistochemical detection of sortilin are shown for (**A**) grade 1, (**B**) grade 2, (**C**) grade 3, and (**D**) GBM. (**E**) Digital quantification of sortilin staining intensities according to grouped pathological subtypes: grade 1–3 (h-score = 4.87, IQR 2.24–13.66 *p* = 0.0016) and GBM (h-score = 22.19, IQR 11.52–36.01). Higher-magnification IHC staining pictures are shown in bottom right magnified insert; scale bar = 30 µm. Data are expressed as individual values with medians. Sortilin h-score median difference between grades 1–3 and GBM was analysed using Mann–Whitney statistical test. (**F**) ROC analysis for sortilin in GBM patient samples. (**G**) Kaplan–Meier survival analysis for patients with low staining (≤median h-score) and high staining (>median h-score). Cases with low sortilin h-score had longer median survival (18 months) compared to high sortilin h-score (12 months) (*p* = 0.0157). ROC: receiver operating characteristic.

Immunohistochemical staining of sortilin was performed on all tissue samples obtained from GBM patients (71 cases) and lower-grade (1–3) glioma (20 cases); the results are presented in Figure 2 and Table 1. Sortilin staining intensity in grade 1–2 glioma was observed to be low in all cases (Figure 2A,B and Table 2), and ten out of twelve cases of grade 3 were observed to have low-intensity staining for sortilin (Figure 2C and Table 2). Sortilin protein expression was higher in GBM than in lower-grade glioma (Figure 2D). Visual observation was confirmed by digital quantification of sortilin staining intensity (Figure 2E), with high sortilin expression in GBM (median h-score = 22.19, IQR 11.52–36.01) compared to glioma grades 1–3 (median h-score = 4.87, IQR 2.24–13.66 *p* = 0.0016). The receiver operating characteristic (ROC) curve [26] indicated an area under the curve (AUC) of 0.81 (Figure 2F). Patient survival data on all glioma and GBM cases revealed that patients with low sortilin had longer survival, with a median survival of 18 months, compared to those with high sortilin, who had median survival of only 12 months (*p* = 0.0157) (Figure 2G). While the survival data for grade 1–3 (Figure S1) and GBM (Figure S2) show no significant difference, there was an observed trend of low sortilin levels associated with longer survival times (13 months and 1.5 months, respectively).

### 3.3. Sortilin Is Detectable in the Plasma of GBM Patients

**Figure 3:**
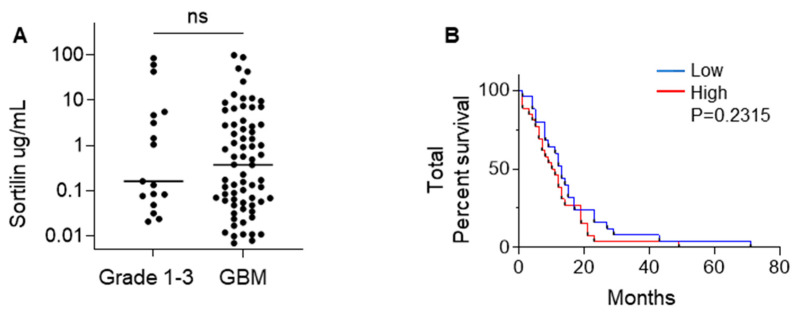
Sortilin plasma quantification in GBM vs. grade 1–3 gliomas. (**A**) Quantitation of circulating sortilin was obtained by ELISA in plasma from grade 1–3 vs. GBM patients. Sortilin plasma quantification is in µg/mL. The median sortilin concentration was 0.163 µg/mL (IQR 0.063 µg/mL–5.136 µg/mL) in combined grades 1–3 versus 0.377 ug/mL (IQR 0.057 µg/mL–2.907 µg/mL) in GBM. Mann–Whitney test was used to evaluate the sortilin concentration median difference between grades 1–3 and GBM. (**B**) Total patient survival based on low (≤median) and high (>median) sortilin concentration in plasma. Cases with low sortilin concentration had longer median survival (13 months) compared to cases with high sortilin concentration (10.5 months).

We measured the circulating concentration of sortilin in the plasma of GBM patients versus glioma grade 1–3 patients by ELISA; the results are reported in Figure 3 and Table 3. Sortilin was detected in all plasma samples at varying concentrations in both GBM and glioma grades 1–3 (Figure 3A). Grades 1–3 sortilin plasma concentrations (median = 0.163 ug/mL, IQR 0.063 ug/mL–5.136 ug/mL) were observed to be lower than GBM sortilin plasma concentrations (median = 0.377 ug/mL, IQR 0.057 ug/mL–2.91 ug/mL), but the difference was not statistically significant. We then wanted to see if circulating sortilin could be a potential biomarker for GBM. We observed a similar pattern to our findings in IHC sortilin-stained tissue samples, with low circulating sortilin concentrations corresponding to longer survival time (low sortilin = 13 months vs. high sortilin 10.5 months); however, this did not meet statistical significance (*p* = 0.2315) (Figure 3B). Interestingly, there was an association between sortilin tissue h-score and sortilin plasma concentration (Table 4); however, there was no significant correlation between tissue sortilin and sortilin plasma concentration (Figure S3A,B).

### 3.4. Sortilin Overexpression in Patient-Derived GBM Cell Lines

**Figure 4:**
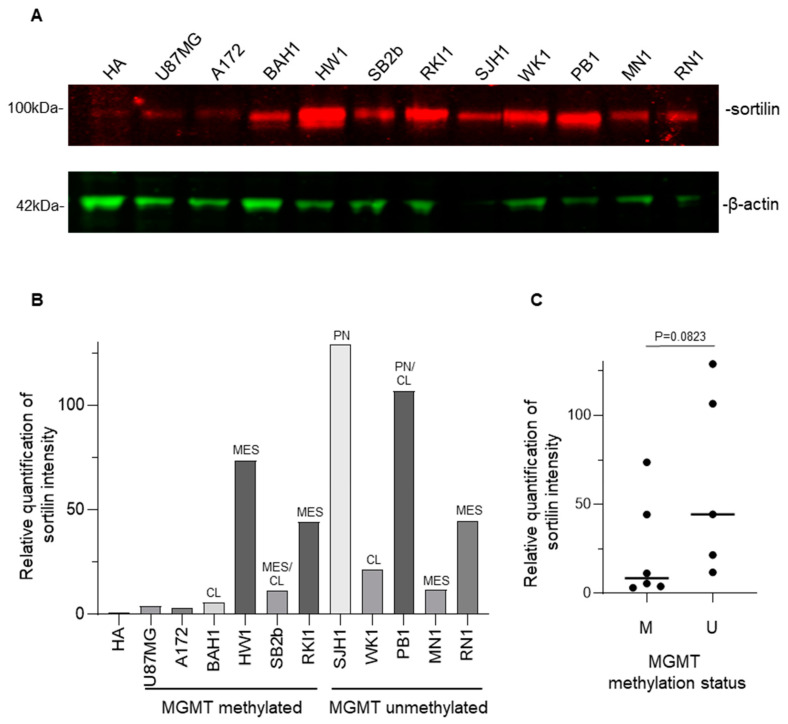
Western blot detection of sortilin in patient-derived GBM cell lines. (**A**) Sortilin was detected as a 100 kDa band (expected molecular mass) in every GBM cell line. The uncropped blots are shown in Figure S6. (**B**) Densitometry analysis of the Western blot. (**C**) Comparison of sortilin expression between GBM cell line, MGMT-methylated (M) and unmethylated (U) MGMT methylation status. Mann–Whitney test was used. Molecular subtypes: CL (classical), MES (mesenchymal), PN (proneural).

Western blot analysis (Figure 4A) was used to detect sortilin expression in human GBM cell lines. Sortilin was observed at the expected molecular weight of 100 kDa in all GBM and HA cell lines (Figure 4A). Densitometric analysis revealed that all GBM cell lines expressed higher levels of sortilin than the control HA cells (Figure 4B). Interestingly, two patient-derived GBM cell lines exhibiting the highest sortilin expression (SJH1 and PB1) were both MGMT-unmethylated and proneural subtype (Figure 4B). GBM cells with unmethylated MGMT seemed to express higher sortilin than GBM cells with methylated-MGMT status (Figure 4C); however, this did not meet statistical significance (*p* = 0.0823). Additionally, when we separated nine patient-derived GBM cell lines by their subtype [21,27], GBM cells with a proneural subtype had significant higher sortilin expression than the classical and mesenchymal subtype (*p* = 0.0091 and *p* = 0.0131, respectively) (Figure S4).

### 3.5. Targeting Sortilin with Small Molecule Inhibitor AF38469 Inhibits GBM Cell Invasion

**Figure 5:**
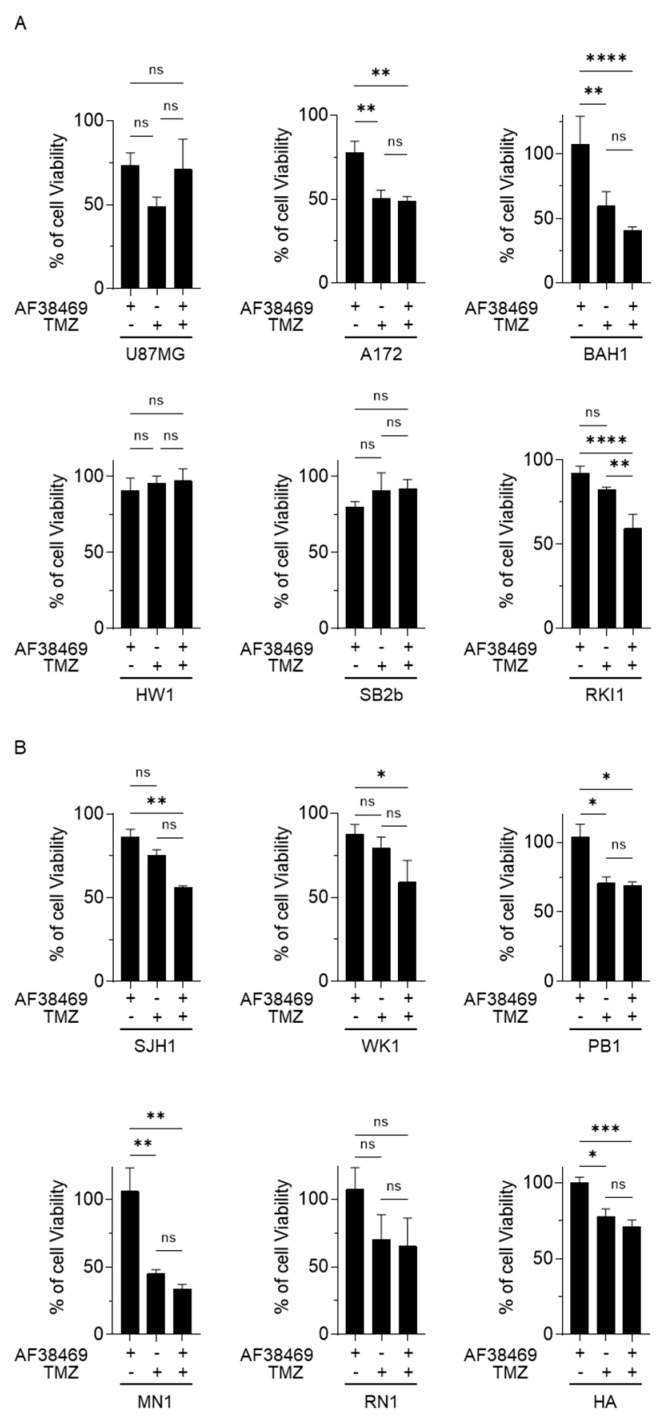
Sortilin targeting effect in GBM cell growth assay. Cell growth was investigated in GBM cells with methylated-MGMT status (**A**) and unmethylated-MGMT status (**B**). Human astrocytes (HA) were used as a control. Treatments were conducted with AF38469 (400 nM), a small molecule inhibitor of sortilin, TMZ (50 µM) and AF38469 in association with TMZ for a 72 h duration. Cell lines treated with TMZ and/or AF38469 were compared to vehicle control (DMSO) treated cells, with 100% viability being the viability of vehicle control (DMSO) treated cells. Data are represented as a mean +/− standard deviation (SD) of three independent experiments. For statistical significance, Student’s *t*-test or ANOVA was used. * *p* < 0.05, ** *p* < 0.01, *** *p* < 0.001, **** *p* < 0.0001, ns: No Significance. TMZ: Temozolomide.

**Figure 6:**
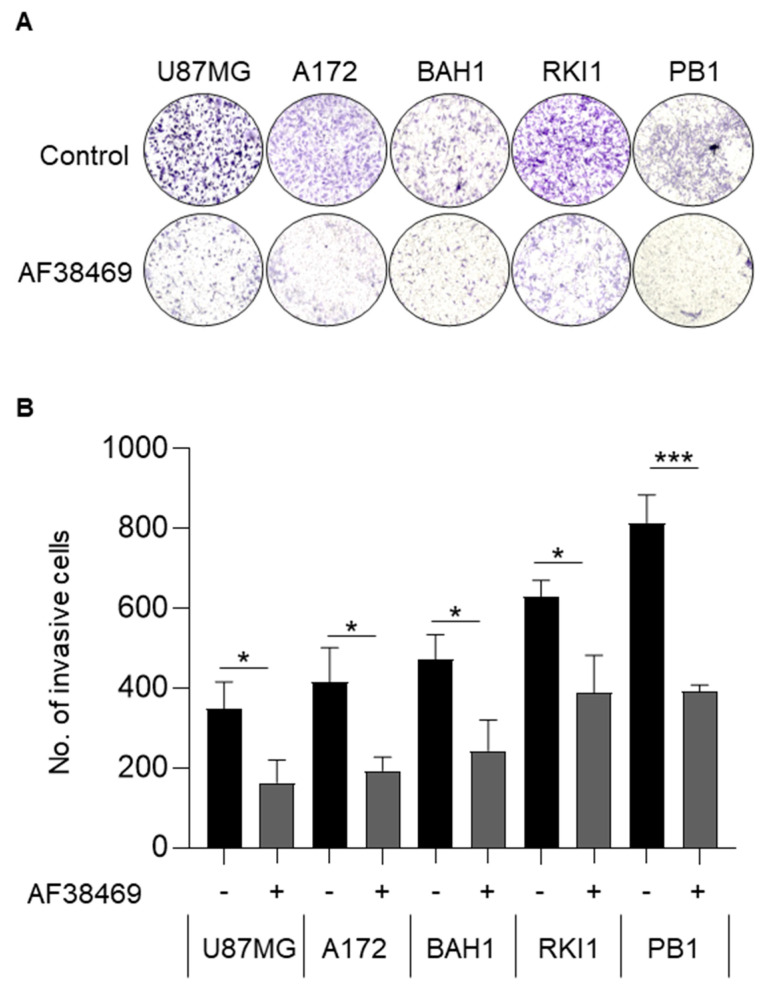
Inhibition of sortilin reduces GBM cell invasion. Cell invasion was investigated using Transwell assay. (**A**) Representative Transwell invasion assay images of untreated GBM cells (top) and GBM cells treated with AF38469 400 nM (bottom). The entire insert with invading cells was observed after crystal violet staining. (**B**) Average number of invading cells treated with AF34869 400 nM (+) compared to untreated (−). After AF38469 treatment there was a 53% reduction of cell invasion in U87MG (*p* = 0.0227), 53% in A172 (*p* = 0.0140), 48% in BAH1, 38% in RKI1 (*p* = 0.0150), and 52% in PB1 (*p* = 0.0005). Cell lines U87MG and A172 were treated for 24 h; BAH1, RKI1 and PB1 were treated for 72 h. Student’s *t*-test was used and the data are the mean +/− standard deviation (SD) of three independent experiments. * *p* < 0.05, *** *p* < 0.001.

We then wanted to test if targeting sortilin using the small molecular inhibitor AF38469 could reduce GBM cell viability and increase the sensitivity to TMZ treatment. Treatment with AF38469 alone did not reduce cell viability in any of the GBM cell lines (Figure 5A,B and Figure S5), suggesting that sortilin is not involved in cell proliferation. However, when combined with TMZ, AF38469 did reduce the cell viability in one of the patient-derived cell lines RKI1 (*p* = 0.0039) (Figure 5A) compared to treatment with TMZ alone. Whether GBM cell lines were MGMT-methylated (Figure 5A) or MGMT-unmethylated (Figure 5B) did not appear to affect AF38469 and TMZ interaction. To further investigate the effect of sortilin inhibition, Transwell invasion assays were performed. Crystal violet staining of invaded cells showed a decrease in the proportion of invaded cells after sortilin inhibition with AF38469 (Figure 6A). In the AF38469-treated GBM cell lines there was a 53% reduction of cell invasion in U87MG (*p* = 0.0227), 53% in A172 (*p* = 0.0140), 48% in BAH1, 38% in RKI1 (*p* = 0.0150), and 52% in PB1 (*p* = 0.0005) (Figure 6B). These results, based on the pharmacological inhibition of sortilin, show that sortilin is necessary for GBM cell invasion and targetable using AF38469.

## 4. Discussion

The heterogeneity of GBM is complex and remains to be fully elucidated, not only to identify new effective targets for treatment, but also to identify noninvasive biomarkers that could enable accurate prognosis and help formulate appropriate treatments. The current study expands on previous observations of the expression and biological effects of sortilin in GBM [14,28] and further examines its potential as a clinical biomarker. The novelty of the present study is related to the following four points. First, we used a larger cohort of patient GBM cases compared to previous studies. Second, we used more recently obtained patient-derived cell lines, which are more clinically relevant. Third, we are the first to include the combined treatment of temozolomide and AF38469. And fourth, we report, for the first time, circulating sortilin in the blood of GBM patients.

In terms of gene expression, our data mining, using GEPIA2 to access the TCGA GBM and LGG datasets, contradicted other studies [14] and revealed no real difference between GBM, LGG and normal tissue expressing SORT1 mRNA. Interestingly, there was a significant difference in the survival outcome for LGG patients between those with high and low SORT1 mRNA expression. Patients with low SORT1 expression had longer survival (overall and disease-free) than patients with high SORT1 expression. However, it cannot be assumed that mRNA abundance equates to protein synthesis [9,29], and therefore it is essential to analyse the protein level directly.

The upregulation of sortilin protein has been reported in several cancers, including breast cancer [30], pancreatic cancer [9], and digestive cancers [6]. In the present study, we show higher sortilin protein expression in GBM compared to lower-grade glioma, and furthermore we show that a high sortilin protein level in tissue is associated with poor survival outcomes. Normal brain tissues were not included in this study, as it is not commonly excised during the surgical removal of the tumor to limit neurological complications in patients. Furthermore, due to the infiltrative nature of GBM, surrounding “normal” adjacent tissue could not be used as a proper control. Normal brain tissue samples could be obtained from cadavers, but we think such samples cannot be compared and are not appropriate controls for GBM biopsies. Therefore, we could not quantify sortilin in the adjacent normal or peritumoral region. In future studies, the peritumoral region would be an area worth exploring, as previous studies have reported that this region contains highly infiltrative cancer stem cells (CSC) [31].

Our study is the first to quantify soluble sortilin in the plasma of GBM patients. Outside GBM, soluble sortilin has already been reported and is presumably formed after proteolytic cleavage [32]. The ELISA used in this study to quantify sortilin was developed based on polyclonal antibodies raised against the extracellular domain (amino acids 78–765) of human sortilin. As such, the ELISA recognizes both the full-length and the cleaved sortilin, and does not discriminate between the two. The potential role of soluble sortilin remains to be elucidated, and while our study reported no significant difference in plasma sortilin concentrations between the glioma and GBM, there was a wide spread of sortilin plasma concentration. There was no correlation between plasma sortilin concentration and GBM tissue sortilin expression, but, interestingly, high concentrations of sortilin were significantly associated with high sortilin tissue expression, suggesting that a significant fraction of circulating sortilin originated from GBM. Other non-cancer studies have reported soluble sortilin as a potential biomarker for cardiac disease [33,34], and, interestingly, these non-cancer studies show a similar spread of concentrations to what we saw in this study, ranging from < 1 ug/mL to 100 ug/mL. The biological and clinical significance of circulating sortilin in GBM is unclear and warrants further investigations in normal and, in particular, GBM cell lines, and further analyses of larger clinical cohort are needed to clarify the clinical biomarker value of sortilin expression (both in tissue and plasma) in GBM.

Aside from tissue expression and plasma release of sortilin in GBM, an interesting finding of the present study was the inhibition of GBM cell invasion induced by sortilin targeting. Sortilin targeting by knockdown and inhibition with AF38469 has previously been shown to reduce cancer cell invasion in a few GBM cell lines. Here, we confirm that AF38469 not only reduces cell invasion in the traditional GBM cell lines (U87MG & A172), but, importantly, also reduces cell invasion in the more recently developed patient-derived cell lines (BAH1, RKI1 and PB1). In terms of GBM treatment, the use of a drug like AF38469 is more clinically relevant than a molecular knockdown, and AF38469 is the only blood-brain-barrier-crossing drug currently available to target sortilin. It is noteworthy to mention that the main issue with GBM recurrence is the invasive capacity of GBM cells, which can invade the brain tissue surrounding the tumor and thus escape surgery or radiotherapy. GBM rarely metastasises, and the main issue is the local invasion of GBM cells in the neural tissue. Interestingly, our data show that the pharmacological inhibition of sortilin, using the orally available blood-brain-barrier-crossing drug AF38469, resulted in the inhibition of patient-derived GBM cell lines. A statistically significant inhibition of GBM cell invasion by AF38469 was observed for tested patient-derived GBM cells, indicating that sortilin is a potential therapeutic target for inhibiting GBM invasiveness. In contrast, sortilin targeting with AF38469 was not found to have any effect on GBM cell survival, and was not found to potentiate the activity of TMZ. However, in one patient-derived cell line (RKI1), potentiation of the cytotoxic effect of TMZ by AF38469 was observed. It is unclear at this stage why RKI1 shows an increased sensitivity to TMZ in presence to AF38469, and this leaves open the possibility that in some GBM, sortilin inhibition could be a way to potentiate the efficacy of TMZ.

It is important to note that the mechanism of action for AF38469 is currently not clearly understood. The structural data published by Schrøder et al. [35] show that AF38469 can interfere with neurotensin binding, therefore preventing this ligand from engaging the sortilin receptor. While this interaction could partially explain the effect of AF38469 on the motility of GBM cell lines, the sortilin receptor has a plethora of additional functions that are independent of neurotensin binding. It is currently unclear whether (and to what degree) these functions are also impacted by AF38469. Furthermore, sortilin does not function as a primary receptor for neurotensin. This role is fulfilled by a different receptor (i.e., the high-affinity receptor for neurotensin or NTSR1), which is another molecule that is highly expressed in GBM [36]. More mechanistic studies are needed for a clear understanding of how sortilin and AF38469 work at the molecular level and how they mechanistically impact the motility of GBM cells. In any case, the finding reported here that sortilin targeting can decrease GBM cell invasion (and eventually proliferation in a limited fraction of GBM) warrants further in vivo investigation.

## 5. Conclusions

In conclusion, the present study points to sortilin as a new potential clinical biomarker for predicting GBM aggressiveness and patient survival. In addition, targeting sortilin seems to decrease GBM cell invasion, and could therefore be used in GBM therapy. Although further in vivo studies are needed, it should be noted that sortilin targeting in human cancers has recently entered a clinical trial phase. In breast [30], thyroid [37] and ovarian cancer [38], targeting sortilin has been shown to enhance the effect of existing chemotherapy by exploiting sortilin function as a receptor allowing targeted entry of a peptide conjugated to docetaxel (TH1902) [10,38,39,40]. There is currently a clinical trial underway targeting sortilin in patients with advanced solid tumors using TH1902 (NCT04706962). This phase 1 clinical trial will include patients with solid tumors in the breast, ovary, endometrium, skin, thyroid, lung, and prostate, and our findings suggest that GBM should be added to the list of cancers that could incorporate sortilin targeting as a therapeutic option.

## Figures and Tables

**Table 1:** Patient clinical information.

Characteristic	Subgroup	Total
Participants	n	91
Sex	Female	37 (40%)
	Male	55 (60%)
Age at diagnosis	Median (min, max)	63 (17, 82)
	Median (Q1, Q3)	63 (56.5, 72)
Grade	1	2 (2.2%)
	2	6 (6.6%)
	3	12 (13.2%)
	GBM	71 (78%)
Tumor site	Frontal	39 (42%)
	Temporal	30 (33%)
	Parietal	15 (16%)
	Other	8 (9%)

**Table 2:** Association between sortilin expression and clinicopathological parameters in glioma.

Parameter	Sortilin Intensity		*p*-Value
	Low	High	
Sex			0.5219
Female	16 (44%)	20 (56%)	
Male	29 (53%)	26 (47%)	
Age			0.4043
≤63	25 (54%)	21 (46%)	
>63	20 (44%)	25 (56%)	
Grade			**<0.0001**
1–3	17 (85%)	3 (15%)	
GBM	28 (39%)	43 (61%)	
Tumor site			0.7241
Frontal	21 (54%)	18 (46%)	
Temporal	14 (48%)	15 (52%)	
Other	10 (43%)	13 (57%)	

The intensity of immunohistochemical staining was categorized as low-staining (≤median H-score) or high-staining (>median H-score). Statistical associations were investigated with Chi-squared test; *p* values of statistical significance (<0.05) are in bold.

**Table 3:** Comparison of sortilin plasma concentration and clinicopathological parameters in glioma.

Parameter	Sortilin Conc.		*p*-Value
	Low	High	
Sex			>0.9999
Female	18 (51%)	17 (49%)	
Male	26 (50%)	26 (50%	
Age			0.6700
≤63	23 (53%)	20 (47%)	
>63	21 (48%)	23 (52%)	
Grade			0.6927
1–3	8 (47%)	9 (53%)	
GBM	35 (50%)	35 (50%)	
Tumor site			0.6622
Frontal	20 (56%)	16 (44%)	
Temporal	14 (50%)	14 (50%)	
Other	10 (43%)	13 (57%)	

Sortilin was assayed by ELISA and categorized as low concentration (≤median µg/mL) vs. high concentration (>median µg/mL). Statistical associations were analysed using Chi-squared test; *p* values of statistical significance (<0.05).

**Table 4:** Association between sortilin concentration in plasma and IHC H-score.

Parameter	Sortilin Conc.		Total	*p*-Value
	Low	High		
Sortilin H-score				
Low	23 (55%)	19 (45%)	87	**<0.0001**
High	5 (11%)	40 (89%)		

Statistical associations were analysed using Chi-squared test; *p* values of statistical significance (<0.05) are in bold.

## Data Availability

The data presented in this study are available on request to the corresponding author.

## Supplementary Materials

The following supporting information can be downloaded at https://www.mdpi.com/article/10.3390/cancers15092514/s1, Figure S1. (A) Grade 1–3 patient survival based on low staining (≤median h-score) and high staining (>median h-score). Cases with low sortilin h-score had longer median survival (36.5 months) compared to high sortilin h-score (23.5 months). (B) GBM patient survival based on low staining (≤median h-score) and high staining (>median h-score). Cases with low sortilin h-score had longer median survival (12.5 months) compared to high sortilin h-score (11 months); Figure S2. (A) Grade 1–3 patient survival based on low (≤median) and high (>median) sortilin concentration in plasma. Cases with low sortilin concentration had longer median survival (35 months) compared to high sortilin concentration (25 months). (B) GBM patient survival based on low (≤median) and high (>median) concentration in plasma. Cases with low sortilin concentration had longer median survival (12 months) compared to high sortilin concentration (10 months); Figure S3. (A) Correlation of sortilin in tissue cytonuclear h-score and soluble sortilin in plasma concentration. (B) Correlation of high sortilin expression in tissue cytonuclear h-score and high soluble sortilin concentration in plasma; Figure S4. Densitometric analysis of sortilin protein intensity by subgroups. CL = classic, MES = Mesenchymal, PN = Proneural. * *p* < 0.05, ** *p* < 0.01, Student’s *t*-test or ANOVA; Figure S5. Viability assay Cell lines treated with AF38469 MGMT methylated v’s MGMT unmethylated; Figure S6. The uncropped blots for Figure 4A.
