# Supplementary material for: The Membrane Protein Sortilin Is a Potential Biomarker and Target for Glioblastoma
Source: Cancers (Basel). 2023 Apr 27;15(9):2514. doi: 10.3390/cancers15092514 (PMC10177035; doi:10.3390/cancers15092514)
Supplement: Supplementary file 1 [file cancers-15-02514-s001.zip › cancers-2339365-supplementary.pdf]

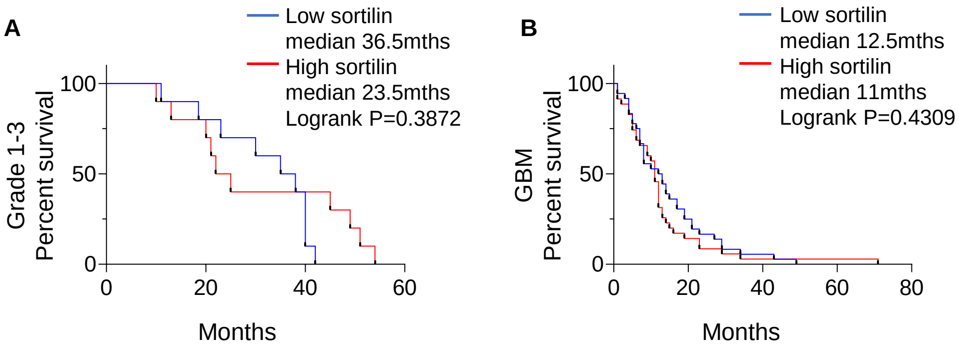

Figure S1. (A) Grade 1-3 patient survival based on low staining (≤median h-score) and high staining (>median h-score). Cases with low sortilin h-score had longer median survival (36.5 months) compared to high sortilin h-score (23.5 months). (B) GBM patient survival based on low staining (≤median h-score) and high staining (>median h-score). Cases with low sortilin h-score had longer median survival (12.5 months) compared to high sortilin h-score (11 months).

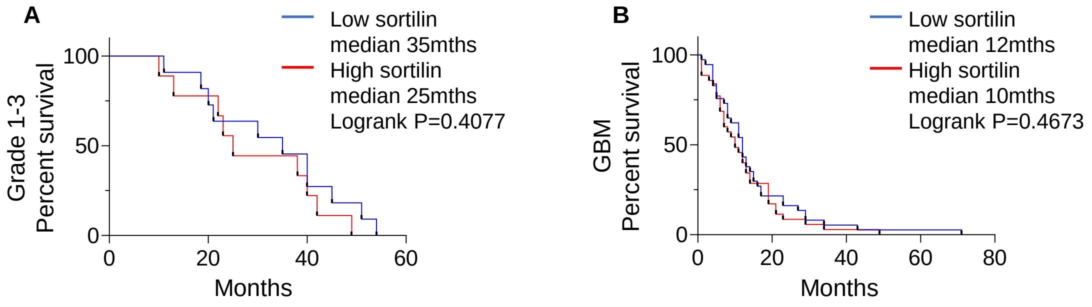

Figure S2. (A) Grade 1-3 patient survival based on low (≤median) and high (>median) sortilin concentration in plasma. Cases with low sortilin concentration had longer median survival (35 months) compared to high sortilin concentration (25 months). (B) GBM patient survival based on low (≤median) and high (>median) concentration in plasma. Cases with low sortilin concentration had longer median survival (12 months) compared to high sortilin concentration (10 months).

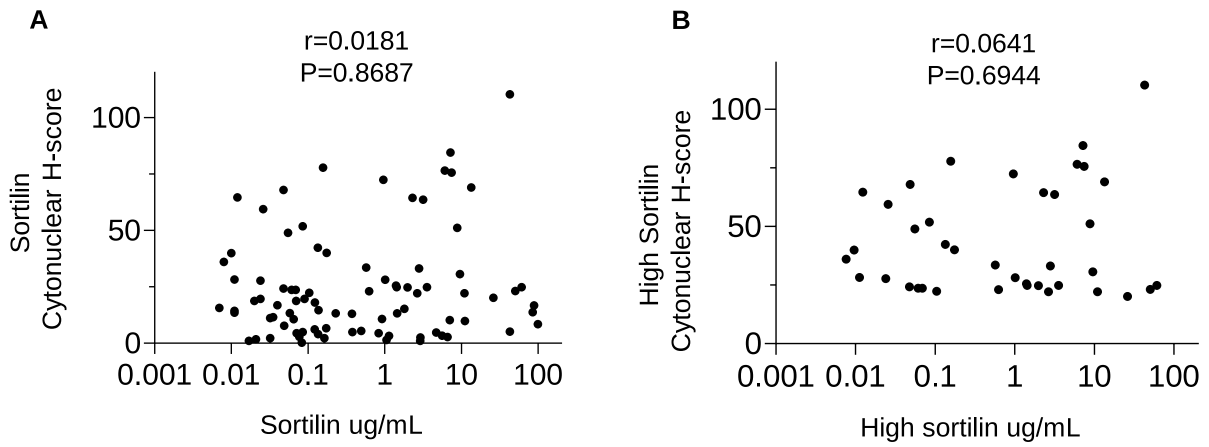

Figure S3. A) Correlation of sortilin in tissue cytonuclear H-score and soluble sortilin in plasma concentration. **B)** Correlation of high sortilin expression in tissue cytonuclear H-score and high soluble sortilin concentration in plasma.

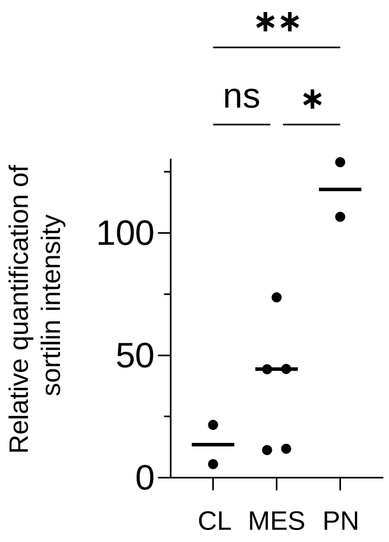

Figure S4. Densitometric analysis of sortilin protein intensity by subgroups. CL = classic, MES = Messenchymal, PN = Proneural. * P<0.05, ** P<0.01, Student’s t-test or ANOVA.

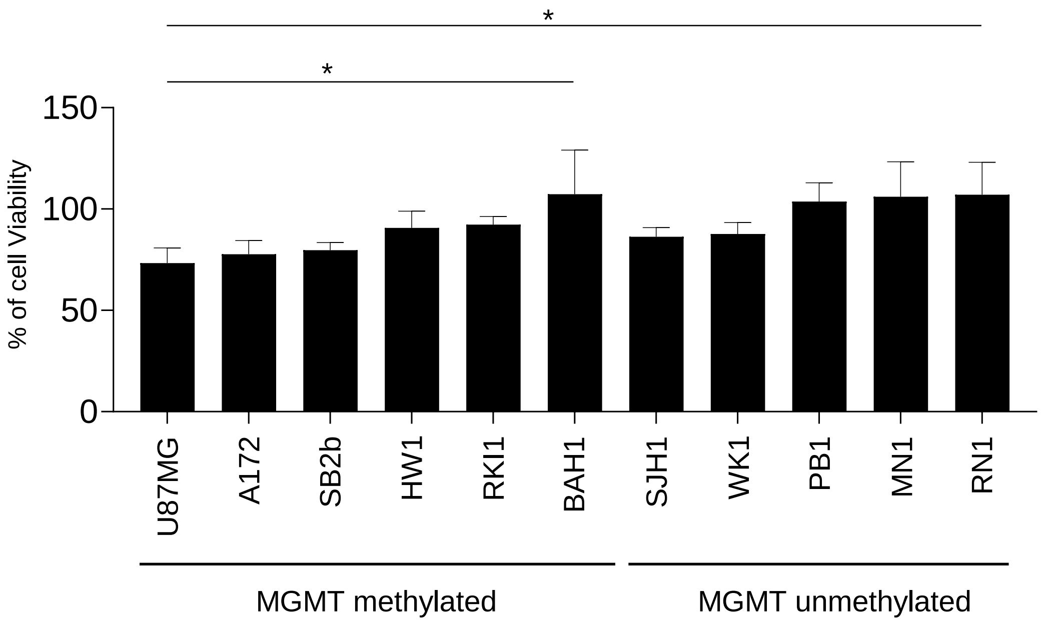

Figure S5. Viability assay Cell lines treated with AF38469 MGMT methylated v's MGMT unmethylated.
