# Supplementary figures and images for: The Membrane Protein Sortilin Is a Potential Biomarker and Target for Glioblastoma
Source: Cancers (Basel). 2023 Apr 27;15(9):2514. doi: 10.3390/cancers15092514 (PMC10177035; doi:10.3390/cancers15092514)

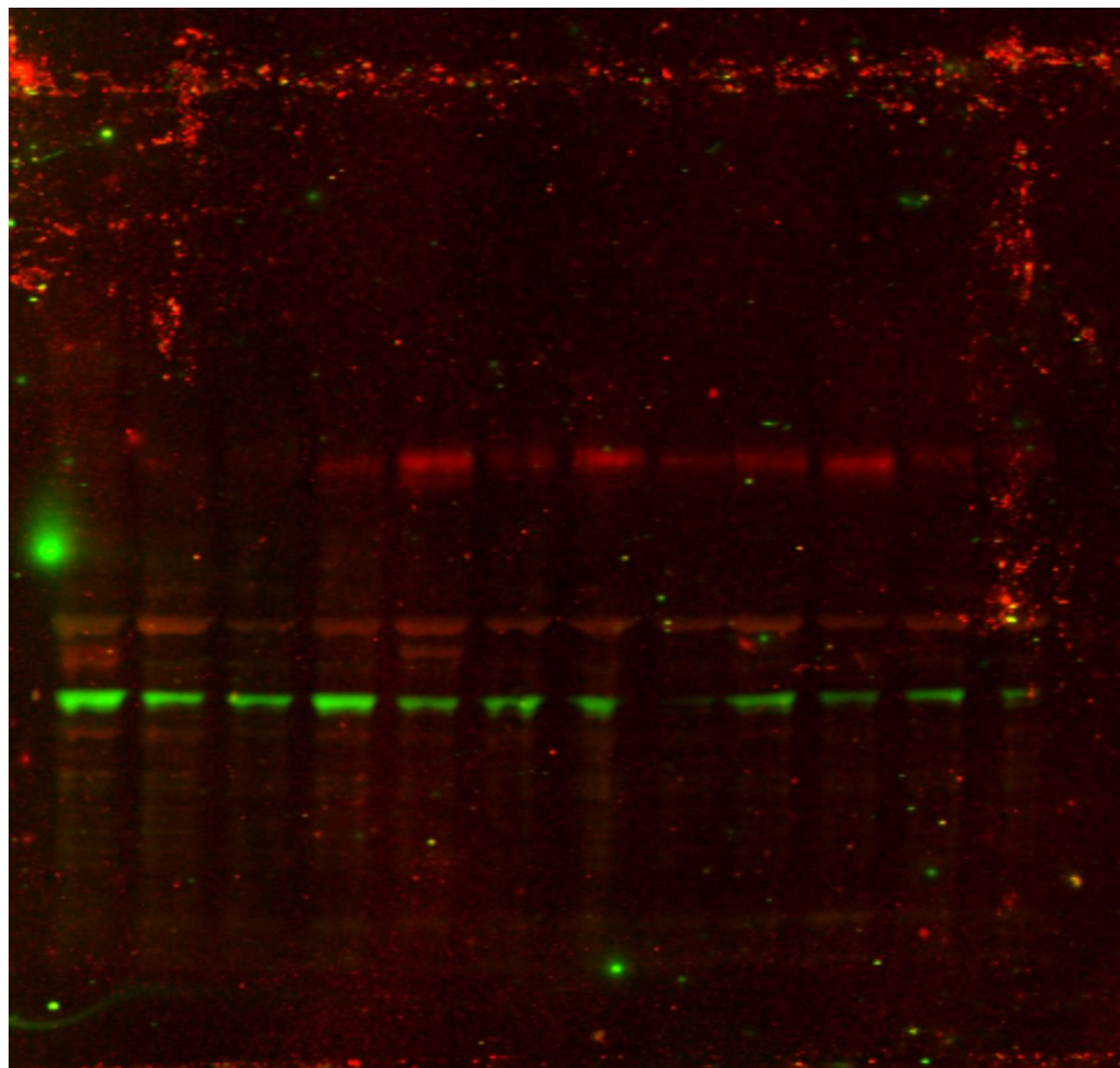

Supplement: Supplementary file 1 [file cancers-15-02514-s001.zip › cancers-2339365-Figure S6.pdf]
